# Parvalbumin interneuron-derived tissue-type plasminogen activator shapes perineuronal net structure

**DOI:** 10.1186/s12915-022-01419-8

**Published:** 2022-10-05

**Authors:** Matthieu Lépine, Sara Douceau, Gabrielle Devienne, Paul Prunotto, Sophie Lenoir, Caroline Regnauld, Elsa Pouettre, Juliette Piquet, Laurent Lebouvier, Yannick Hommet, Eric Maubert, Véronique Agin, Bertrand Lambolez, Bruno Cauli, Carine Ali, Denis Vivien

**Affiliations:** 1 Normandie Univ, UNICAEN, INSERM, INSERM UMR-S U1237, Physiopathology and Imaging of Neurological Disorders, Institut Blood and Brain @ Caen Normandie, Cyceron, Bd Becquerel, BP 5229-14074, 14000 Caen, France; 2 Neuroscience Paris Seine - Institut de Biologie Paris Seine (NPS - IBPS), Sorbonne Université UM119, CNRS UMR8246, INSERM U1130, 75005 Paris, France; 3 Department of clinical research, CHU de Caen Normandie, Caen, France

**Keywords:** Tissue-type plasminogen activator, Plasminogen, Parvalbumin interneurons, Perineuronal nets, Aggrecan

## Abstract

**Background:**

Perineuronal nets (PNNs) are specialized extracellular matrix structures mainly found around fast-spiking parvalbumin (FS-PV) interneurons. In the adult, their degradation alters FS-PV-driven functions, such as brain plasticity and memory, and altered PNN structures have been found in neurodevelopmental and central nervous system disorders such as Alzheimer’s disease, leading to interest in identifying targets able to modify or participate in PNN metabolism. The serine protease tissue-type plasminogen activator (tPA) plays multifaceted roles in brain pathophysiology. However, its cellular expression profile in the brain remains unclear and a possible role in matrix plasticity through PNN remodeling has never been investigated.

**Result:**

By combining a GFP reporter approach, immunohistology, electrophysiology, and single-cell RT-PCR, we discovered that cortical FS-PV interneurons are a source of tPA in vivo. We found that mice specifically lacking tPA in FS-PV interneurons display denser PNNs in the somatosensory cortex, suggesting a role for tPA from FS-PV interneurons in PNN remodeling. In vitro analyses in primary cultures of mouse interneurons also showed that tPA converts plasminogen into active plasmin, which in turn, directly degrades aggrecan, a major structural chondroitin sulfate proteoglycan (CSPG) in PNNs.

**Conclusions:**

We demonstrate that tPA released from FS-PV interneurons in the central nervous system reduces PNN density through CSPG degradation. The discovery of this tPA-dependent PNN remodeling opens interesting insights into the control of brain plasticity.

**Supplementary Information:**

The online version contains supplementary material available at 10.1186/s12915-022-01419-8.

## Background

Perineuronal nets (PNNs) are a specific form of extracellular matrix (ECM), in which chondroitin sulfate proteoglycans (CSPGs), hyaluronan, link proteins, and tenascin-R are organized into dense lattice-like structures [26, 86]. In the cerebral cortex, PNNs mainly enwrap fast-spiking parvalbumin (FS-PV) interneurons. They are formed during post-natal development and are thought to stabilize neuronal circuits at adult stages [12, 26, 89, 96]. Indeed, PNN digestion with the bacterial enzyme chondroitinase ABC (ChABC) strongly affects neuronal excitability and influences brain plasticity [2, 11, 30, 42, 62, 70, 76]. In the brain, endogenous proteases such as A Disintegrin And Metalloproteinase with Thrombospondin Motifs (ADAMTS) or matrix metalloproteinases (MMPs) may regulate PNN functions through the cleavage of their CSPGs, including aggrecan, neurocan, versican, and brevican [34, 86]. Paradigms of sensory deprivation have demonstrated the critical implication of PNN remodeling during periods of heightened plasticity, as well as the involvement of endogenous MMP9 in PNN plasticity [37, 59, 60, 66]. Interestingly, it has been shown that the expression and activity of the serine protease, tissue-type plasminogen activator (tPA) increase after sensory deprivation. Furthermore, tPA is thought to participate in plasticity-related mechanisms [16, 52, 53, 58]. However, a direct link between tPA-driven plasticity and PNNs remains unestablished.

In the (neuro-)vascular clinical community, tPA is well known as being the major enzymatic activator of plasminogen, thus promoting vascular fibrinolysis. Besides, tPA also has prominent effects in the central nervous system (CNS). As reviewed recently [87], many actions of tPA in the CNS rely on its proteolytic activity. Some functions occur independently of plasmin(ogen), via different receptors and binding partners which include laminin, low-density lipoprotein receptor–related proteins (LRPs), N-methyl-D-aspartate receptor (NMDAR), or some growth factor precursors. Via these multiple targets, tPA is a key player in CNS physiological and pathological processes (for recent reviews see [87, 98]). For instance, during development, tPA controls neuronal migration [64] and promotes axonal growth/synaptic plasticity [4, 67, 68, 82]. Later in life, tPA modulates learning and memory processes [5, 32, 48] and anxiety-related behaviors [54, 65]. Under pathological conditions, tPA controls neuronal death [44, 45, 61, 94], neuro-inflammation, and blood–brain barrier permeability [47, 55, 77, 81].

Consensual descriptions of the cellular distribution of tPA in the CNS are lacking. So far, most literature agrees that tPA can be released in the extracellular space by endothelial cells, oligodendrocytes, and hippocampal neurons [43, 46, 80]. Microglia and astrocytes are uncertain sources of tPA under physiological conditions but might be under pathological conditions [1, 91]. In the CNS, tPA is easily detected (mRNA and protein) in hippocampal mossy fibers [46, 74, 85], but its immunohistological detection in cortical neurons is only available through its somatic accumulation after the blockade of axo-dendritic transport with colchicine. Interestingly, immunohistological (after colchicine injection), electrophysiological, and transcriptional analyses revealed the expression of tPA in a subset of excitatory neurons [46, 84]. tPA expression has also been suggested in some GABAergic interneurons in different brain areas [17, 28, 80].

In the present study, using a viral reporter construct, electrophysiology, and single-cell RT-PCR in mice, we found that cortical GABAergic neurons, mainly FS-PV interneurons, express tPA. Using conditional mice presenting a depletion of tPA restricted to PV cells, as well as primary culture of interneurons, we show that tPA originating from PV cells controls PNN turnover through plasmin. This regulation occurs through the cleavage of a component crucial for PNN structural integrity, the CSPG aggrecan.

## Results

### tPA is expressed by cortical fast-spiking parvalbumin interneurons in vivo

**Fig. 1:**
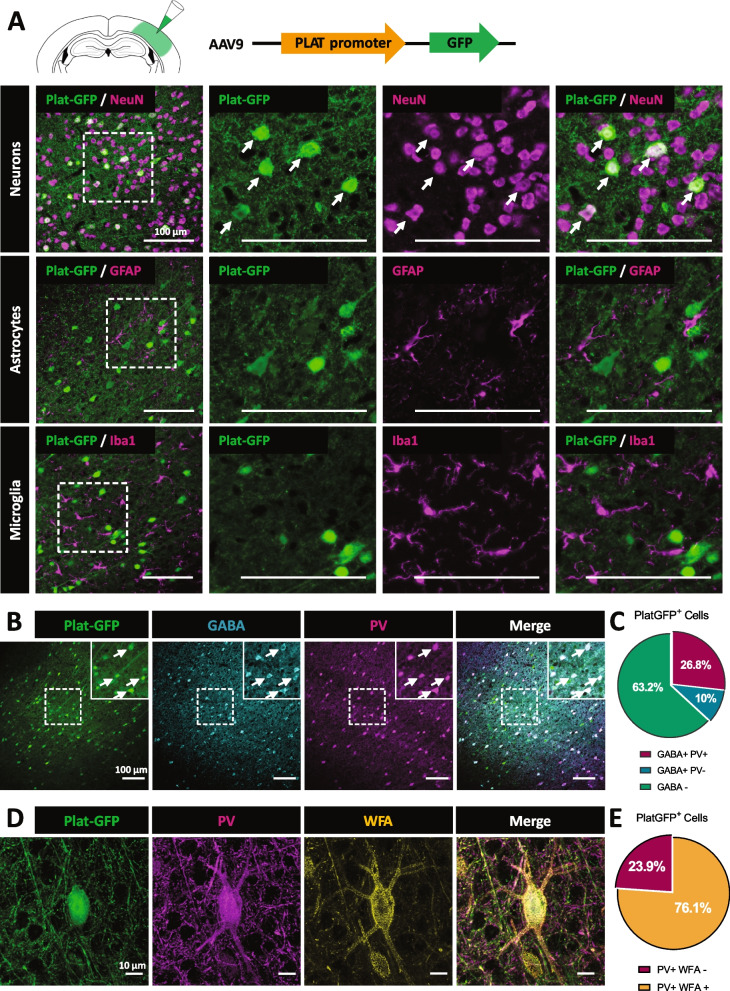
tPA is expressed by parvalbumin interneurons enwrapped with PNNs. **A** AAV-Plat-GFP was injected in the somatosensory cortex of adult wild-type mice. Co-immunohistochemistry of the GFP reporter (green) with the neuronal marker NeuN (magenta), the astrocyte marker GFAP (magenta), and the microglial marker Iba-1 (magenta) in the somatosensory cortex of adult WT mice 3 weeks after AAV injection. AAV-Plat-GFP induces expression of GFP in neurons but not in astrocytes or microglia (Scale bar: 100 μm). **B**,**C** Co-localization of the Plat-GFP reporter (green) with GABA (cyan) and parvalbumin (magenta) markers following injection of AAV-Plat-GFP in the somatosensory cortex of WT mice (Scale bar: 100 μm). Quantitative analysis of Plat-mediated expression of GFP in relation to GABAergic positive neurons and parvalbumin interneurons. **C** Around 37 % of Plat-GFP positive neurons are GABAergic interneurons and 73 % of them co-localized with PV marker (*n*=4 coronal sections from *N*=3 WT brains). **D** 3D reconstruction of a Plat-GFP positive neuron (green), co-labeled with PV (magenta) and the PNN marker Wisteria floribunda agglutinin (WFA, yellow) (Scale bar: 10 μm). **E** Quantification of Plat-GFP neurons double stained with PV and WFA

We sought to identify the cell subtypes expressing tPA in the somatosensory cortex of adult mice. We used a viral reporter construct encoding for green fluorescent protein (GFP) under the control of the human tPA (Plat) promoter (AAV-Plat-GFP, Fig. 1A). This construct was first validated in the dentate gyrus (DG; Additional file 1: Fig. S1A-B), a brain structure in which tPA expression is well described [46, 80]. After injection of the AAV-Plat-GFP in the somatosensory cortex, immunohistochemical analyses revealed that the GFP reporter was only detected in NeuN-positive neuronal cells but neither in GFAP-positive astrocytes nor in Iba-1-positive microglia (Fig. 1A; these three cell types are potential targets of AAV9-driven infection [50, 56]). Even under inflammatory conditions (lipopolysaccharide treatment), neither reactive astrocytes nor reactive microglia expressed GFP, excluding the expression of tPA in these cell types (Additional file 1: Fig. S2).

Confirming our previous findings [46], a subset of tPA-GFP+ neurons were excitatory glutamatergic neurons, as they expressed Tbr1 (Additional file 1: Fig. S3). In addition, we found that 36.9 % of Plat-GFP+ neurons were positive for GABA (Fig. 1B,C). Among these Plat-GFP GABAergic interneurons, the majority (72.7 %) were parvalbumin+ (Fig. 1B,C) and 76.1 % of these parvalbumin+ neurons were enwrapped with PNNs (stained with WFA; Fig. 1D,E). In situ hybridization confirmed the presence of tPA mRNA in PV interneurons, and more precisely those enwrapped by PNNs (Additional file 1: Fig. S4).

**Fig. 2:**
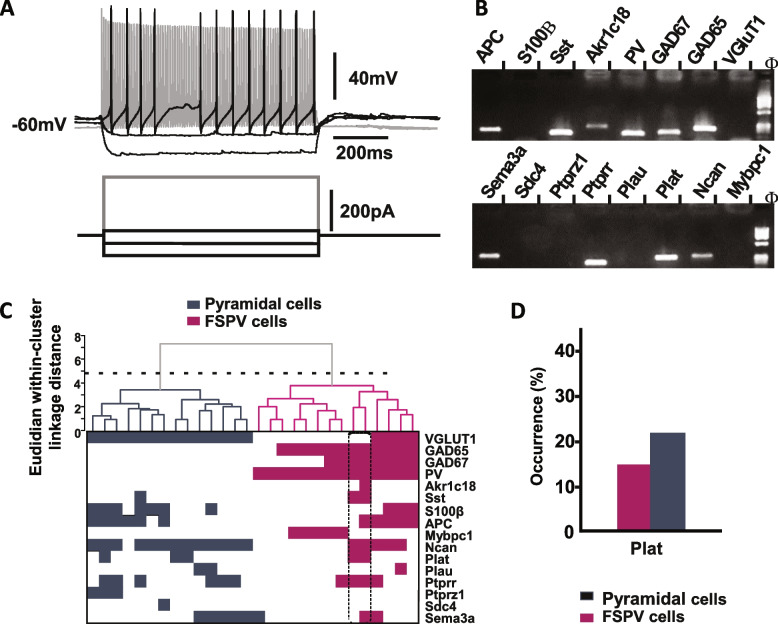
Electrophysiological and molecular characterization of tPA expressing FS-PV interneurons. **A**,**B** Characterization of Plat-expressing FS-PV cells. **A** Current-clamp recordings of a Plat-expressing FS-PV interneuron. Upper traces show voltage responses to current steps (bottom traces). This neuron typically displayed a low input resistance, a high rheobasic current (120 pA, black trace first depolarizing current step) and fired short-duration action potentials with fast and large after hyperpolarizing potentials. A strong depolarizing current (380 pA) evoked a high and sustained firing rate with little or no spike frequency adaptation (shaded trace). **B** Agarose gel analysis of the RT-PCR products of the same FS-PV neuron showing expression of VGlut1, GAD65, GAD67, PV, Akr1c18, Sst, APC, Ncan, Plat, Ptprr, and Sema3a. **C** Ward’s clustering based on the expression of 16 genes performed on 28 cortical neurons (upper panel). The *x*-axis represents individual cells and the *y*-axis the average Euclidian within-cluster linkage distance. Pyramidal (grey) and FS-PV (magenta) cells were segregated into two first-order clusters as suggested by Thorndike procedure (dotted line). Gene expression profile of genes expression across the different cell clusters (bottom panel). For each cell, colored and white squares indicate presence and absence of genes, respectively. Note the presence of VGlut1 in pyramidal cells and of PV in FS-PV cells. Plat-expressing FS-PV neurons (dotted box) displayed a similar gene expression profile and were segregated together. **D** Histograms depicting a similar occurrence of Plat in pyramidal and FS-PV cells

We also performed electrophysiological and molecular characterizations of 28 neurons from deeper layers (IV-V) of the mouse primary visual cortex by combining patch-clamp recordings and single-cell RT-PCR [13]. The PCR protocol was designed to probe the expression of different cell type-specific markers (VGLUT1, GAD65 and GAD67, PV, S100β, Sst, APC, and Akr1c18), plasminogen activators (Plat: tPA gene and Plau: urokinase), and extracellular matrix-related genes (Ncan, Ptptrz1, Ptprr, Sema3a, Sdc4, and Mybpc1; Additional file 1: Table S1). Pyramidal cells (*n*=14) were identified by the triangular shape of their soma and their regular spiking firing pattern characterized by broad spikes and marked frequency adaption. FS-PV interneurons of G42 mice (PV-expressing reporter mouse) [15] were characterized by low input resistance and high rheobase currents [36]. FS-PV interneurons fired short-duration action potentials with sharp post-hyperpolarizing potentials and were able to sustain a high firing rate with little or no spike frequency adaptation (Fig. 2A). The molecular analysis of the FS-PV interneuron shown in Fig. 2B confirmed the expression of PV and GAD65/67, as well as SST, APC, Akr1c18, Plat, Ncan, Ptprr, and Sema3a.

Analyzed neurons were grouped together according to their overall molecular similarity using Ward’s clustering [14], yielding two main groups (Fig. 2C). All neurons in the left cluster were pyramidal cells expressing VGLUT1 but none in either cluster expressed GADs or PV. By contrast, all neurons in the right cluster expressed PV and were identified as FS-PV interneurons. A sub-cluster of two FS-PV interneurons (dotted box, Fig. 2C) displayed a similar molecular profile with expression of Sst, Plat, Ncan, Plat, and Ptprr. Plat mRNA was detected in 14.3 % of FS-PV cells (*n*=2 of 14 cells, Fig. 2D), a proportion similar to that observed in pyramidal cells (21.4 %, *n*=3 of 14 cells, *p*=0.663) and confirming our previous observations [46].

### tPA produced by PV interneurons degrades surrounding PNNs in a plasmin-dependent manner (in vivo and in vitro investigations)

**Fig. 3:**
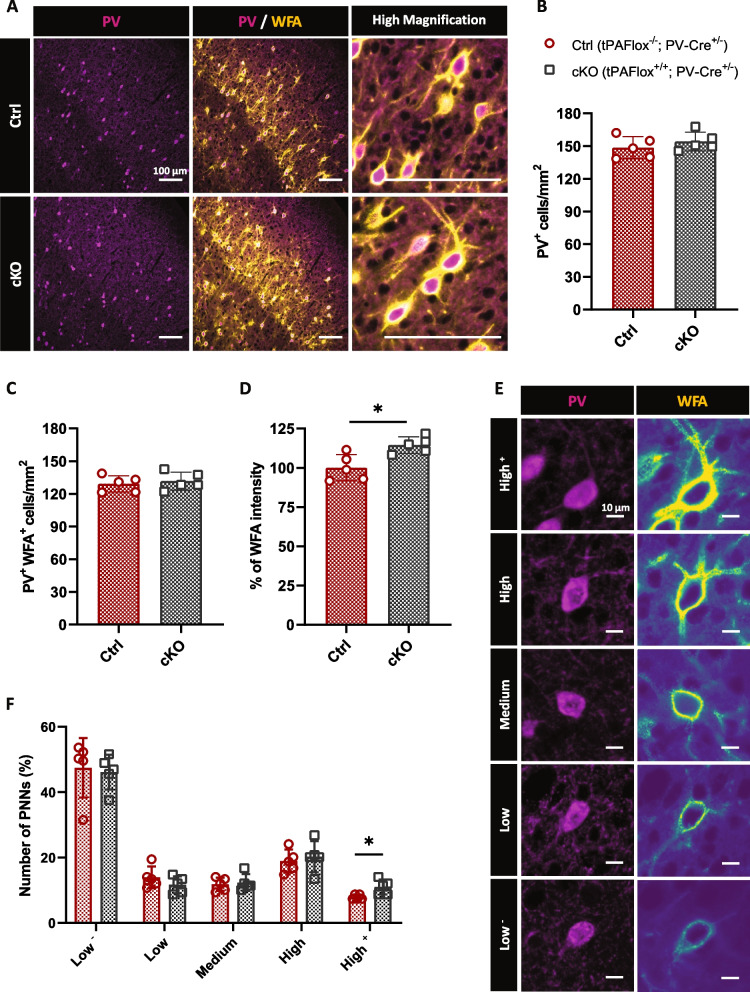
Specific deletion of tPA in PV interneurons affects PNNs morphology. **A** Representative images of PV cells (magenta) and PNNs (yellow) in the somatosensory cortex of adult tPAFlox^+/+^; PV-Cre^+/−^ mice (cKO) and their control littermates (Ctrl), tPAFlox^−/−^; PV-Cre^+/−^ mice (Scale bar: 100 μm). Quantitative analysis of the density of PV cells (**B**) and PNN-containing PV cells (**C**) in both genotypes. **D** Average WFA intensity was quantified in both groups. **E** PNNs were assigned to different categories based on the intensity of WFA staining (Scale bar: 10 μm). The percentage of each PNN subtype (**F**) and the graph show mean ± sem (*N*=5 animals; *n*=4 coronal sections per animal). Mann–Whitney test; *: *p*<0.05

To assess the effect of tPA expressed by PV cells on the remodeling of PNNs, we generated a conditional knockout mouse strain lacking tPA in PV cells by crossing tPAFlox^+/+^ mice with PV-Cre^+/−^ mice (tPAFlox^−/−^ ; PV-Cre^+/−^ mice were used as controls, Additional file 1: Fig. S5). In both genotypes, the total number of PV interneurons, as well as the number of PV interneurons enwrapped with PNNs, was similar (Fig. 3A–C). However, the overall intensity of WFA was higher in cKO mice than in WT mice (+14.4 %, Fig. 3D). A semi-quantitative analysis based on a qualitative grading PNN staining intensity around PV soma, from very low to very high WFA staining (Fig. 3E), revealed a slightly higher percentage of neurons enwrapped with the highest degree of PNN coverage in cKO mice (Fig. 3F). Overall, these observations support the idea that tPA released from PV interneurons can contribute to PNN turnover in vivo.

**Fig. 4:**
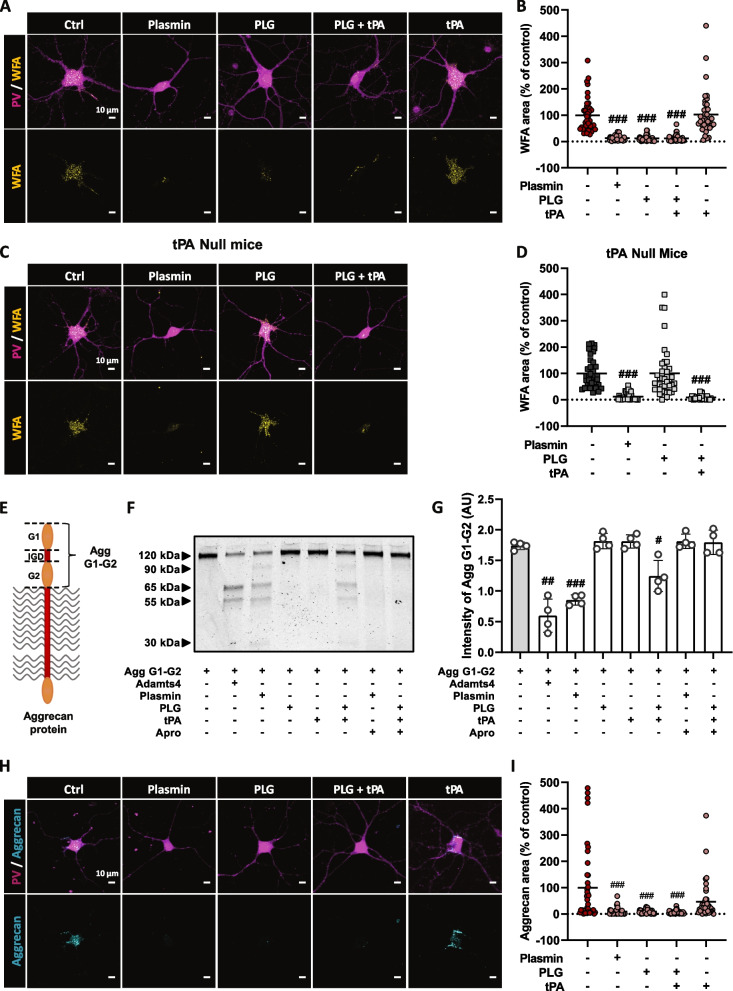
Endogenous tPA controls the remodeling of PNNs through plasmin-dependent aggrecan cleavage. **A**,**B** MGE-derived WT interneuron cultures were treated at DIV14 with plasmin (200 nM), plasminogen (100 nM), tPA (10 and 200 nM), and processed 24h later for immunocytochemistry. **A** Representative images for PV (magenta) and WFA (yellow) stainings. **B** Quantification shows that plasmin, plasminogen, and plasminogen + tPA reduce WFA staining, whereas tPA has no effect. **C** Representative images of MGE-derived tPA Null interneuron cultures stained for PV (magenta) and WFA (yellow) 24h after treatment with plasmin (200 nM), plasminogen (100 nM), and tPA (10 nM). **D** WFA-positive area quantification reveals that plasmin induces the degradation of PNN in PV cells from tPA Null cultures whereas plasminogen fails to (mean ± sem; *n*=40 cells from 4 independent experiments). Kruskall-Wallis test followed by Dunn’s post hoc test for multiple comparisons; ###: *p*<0.001 (compared to control), ***: *p*<0.001 (compared to plasminogen + tPA). Scale bar: 10 μm. **E–G** Human aggrecan G1-G2 (156 nM) was incubated with ADAMTS4 (positive control), plasmin, plasminogen, tPA (7.8 nM for each protease), and aprotinin (78 nM) overnight. **F**,**G** Degradation products were visualized by Imperial blue staining. Like ADAMTS4, plasmin, and plasminogen + tPA can cleave aggrecan G1-G2 in vitro. Aprotinin reverses plasmin-dependent aggrecan degradation. *N*=4 independent experiments. One-way ANOVA, Fisher’s LSD post hoc test; #: *p*<0.05; ##: *p*<0.01; ###: *p*<0.001 (compared to Agg G1-G2). **H** Representative images of MGE-derived WT interneuron cultures treated with plasmin (100 nM), plasminogen (100 nM), and tPA (10 and 200 nM) and co-stained with aggrecan (cyan) and PV (magenta). **I** Quantitative analysis of aggrecan positive area shows that plasmin, plasminogen, and plasminogen + tPA reduce aggrecan staining whereas tPA treatment has no effect in WT interneurons (mean ± sem; *n*=40 cells from 4 independent experiments). Kruskall-Wallis test followed by Dunn’s post hoc test for multiple comparisons; ###: *p*<0.001 (compared to Control). Scale bar: 10 μm

To dissect the potential underlying mechanism of this effect, we used a model of primary murine interneuron cultures ([27]; Fig. 4). Immunocytochemistry confirmed the presence of PNN-like structures (displaying WFA labelling and expressing aggrecan and hyaluronic acid, two major components of PNN; Additional file 1: Fig. S6A) that were sensitive to ChABC (Additional file 1: Fig. S6B). We assessed PNN integrity in response to treatment with tPA alone or in combination with its conventional substrate plasminogen, plasminogen alone, or active plasmin (Fig. 4A,B). After 24h exposure, we observed a significant PNN degradation with plasmin treatment (−87.3%) or with the combination of plasminogen+tPA (−87.6%), while tPA alone did not alter PNN turnover (Fig. 4A,B). This suggests that tPA might not directly promote PNN degradation. Accordingly, aprotinin, a blocker of plasmin proteolytic activity, prevented plasminogen + tPA-induced PNN degradation (Additional file 1: Fig. S7A-B) demonstrating that tPA promotes PNN degradation via a plasmin-dependent mechanism. We found that plasminogen alone could also induce PNN degradation (−88.4 %; Fig. 4A,B), suggesting that endogenous tPA released by interneurons converts extracellular plasminogen into plasmin. To confirm this, a fluorescent assay of plasmin activity revealed the actual conversion of plasminogen in active plasmin in the absence of exogenous tPA treatment (Additional file 1: Fig. S7C-D). We also exposed cultured interneurons from tPA Null mice to plasminogen, plasmin, or plasminogen + tPA during 24 h (Fig. 4C,D). Although plasmin and plasminogen + tPA significantly reduced WFA staining (−88.2 % for plasmin; −91.5 % for plasminogen + tPA), plasminogen alone did not (Fig. 4C,D), confirming that PNN degradation depends on endogenous tPA produced by interneurons. We also found that plasmin-dependent degradation of PNNs in vitro was not sensitive to TIMP3 [90], suggesting that MMPS or ADAMTS are not downstream effectors in this pathway (Additional file 1: Fig. S8). To understand how plasmin promotes PNN degradation, we incubated a fragment of the aggrecan core protein (corresponding to G1-IGD-G2) with plasminogen, plasmin, tPA alone or in combination, and with or without aprotinin (Fig. 4E–G). ADAMTS-4 was used as a positive proteolytic control. Aggrecan core protein was efficiently cleaved by plasmin (51% loss in the signal of the core protein) and plasminogen + tPA (28.7 % loss in the signal of the core protein), leading to two fragments (55 and 65 kDa) which were similarly obtained with the control treatment with ADAMTS4 (−66%). Plasmin treatment also leads to two products of 90 and 30 kDa due to a second cleavage site. Moreover, as observed with WFA staining, a significant decrease of aggrecan staining around PV cells in vitro revealed that tPA-dependent activation of plasminogen degrades this perineuronal CSPG in situ (Fig. 4H,I).

## Discussion

The present study provides the first demonstration that tPA expressed by FS-PV interneurons regulates PNN remodeling, through the conversion of plasminogen into plasmin. This remodeling results from the cleavage of a major component of PNNs, the CSPG aggrecan.

tPA expression was previously reported in excitatory pyramidal neurons using in situ hybridization techniques, immunohistochemistry, and single-cell PCR coupled with patch-clamp recordings [46, 75]. Sparse and inconsistent studies have also reported tPA expression in some subtypes of GABAergic interneurons. Immunohistological studies [28] have reported tPA expression in VIP-positive, but not in SST-positive perivascular interneurons. Using a reporter mouse line, Stevenson and Lawrence discovered the specific expression of tPA in SST-positive oriens-lacunosum moleculare interneurons, a subpopulation of hippocampal interneurons responsible for synaptic plasticity in Shaffer collateral synapses [80]. Furthermore, the presence of tPA in PV-positive neurons but not in SST-positive neurons has been proposed in the somatosensory cortex, although the specificity of the staining is not entirely clear [17]. One of our main findings is the demonstration by molecular, electrophysiological, and single-cell RT-PCR approaches that tPA is expressed by GABAergic interneurons, including FS-PV interneurons. These FS-PV interneurons are fundamental for several behavioral responses and their dysfunction participates in several diseases, including schizophrenia and autism [35, 73]. Interestingly, their activity is at least in part under the control of a specialized ECM that surrounds them, the PNNs. PNNs are dynamic structures, not only during experience-driven plastic developmental periods [51, 83, 96], but also in adults. For instance, modification of PNN density with the diurnal-nocturnal cycle, or their enzymatic degradation may have a potential impact on memory processing [30, 63, 78]. In line with this, memory-related diseases (Alzheimer’s disease, drug addiction), but also many other pathological conditions (schizophrenia, epilepsy, depression, multiple sclerosis, stroke, Huntington’s disease) may to some extent involve or impact PNN integrity and function [12, 23, 86, 93].

CSPGs, the major components of PNNs, are sensitive to endogenous processing by proteases of the MMP and ADAMTS families [34, 86]. FS-PV interneurons and pyramidal cells express an array of proteases that could thus process PNNs from both sides [21, 71]. Previous studies have shown that the tPA/plasmin system can degrade several CSPGs found in PNNs, such as neurocan, versican, and phosphacan [38, 95] and can activate pro-MMPs and pro-ADAMTS4 into their mature form [19, 41]. Interestingly, the tPA/plasmin system can also increase ChABC-mediated axonal regrowth after spinal cord injury through CSPG degradation [9]. However, the direct evidence of PNN degradation by the tPA/plasmin system has never been provided. Here, we show that the conditional deletion of tPA in FS-PV interneurons in vivo leads to a moderate increase in PNN density in the somatosensory cortex, which is indicative of a cell-autonomous regulation of PNN plasticity under physiological conditions. Thus, the histological modifications observed in vivo under basal conditions could reflect modifications of the dynamic balance between CSPG synthesis and their degradation. However, using in vitro experiments, by combining cell imaging and pharmacology, we demonstrate that tPA indeed promotes PNN degradation around PV interneurons through plasminogen conversion into plasmin. The latter can be blocked by aprotinin and is independent of MMP or ADAMTS activation, since a broad-range inhibitor (TIMP3) did not reverse the effects of plasmin. Overall, our study shows that tPA/plasmin can exert a cell-autonomous effect on PNN degradation when expressed by PV interneurons. Moreover, since tPA was described to be induced as an immediate early gene following seizure or long-term potentiation [68], we can hypothesize that its expression may be also triggered in PV cells under certain conditions to promote PNN remodeling. In addition, considering that tPA is also expressed in the brain by various cell types including pyramidal cells [46] or oligodendrocytes [43], it could also probably drive PNN degradation through a non-cell autonomous manner when released in the extracellular space.

The consequence of PNN degradation on FS-PV cell function has been the subject of several studies, sometimes with contradictory results [2, 22, 24, 25, 31]. For instance, PNN digestion induced by chondroitinase ABC was shown to reduce [2] or increase the excitability of interneurons [22]. However, these discrepancies could be explained by the experimental approaches used. Indeed, genetic removal of specific PNN components might affect the development of neuronal networks, which is not the case when PNN are acutely digested in the adult brain. Additionally, in vitro models cannot reflect the complexity of neuronal networks. It would be relevant to perform fine electrophysiological studies to determine the impact of tPA-driven degradation of PNN on PV cell functions. Furthermore, it has been described that the activity of PV cells and the density of PNNs influence fear memory and social behaviors [3, 6, 8, 18, 69, 79]. Therefore, it would also be interesting to investigate the consequence of PNN degradation by the tPA/plasmin, using PV-Cre x tPA flox mice, on these behavioral responses.

Finally, we also demonstrate that tPA-mediated PNN degradation can occur through the cleavage of the core protein of aggrecan. However, we cannot exclude that the plasminogen activator system may also promote PNN degradation through the cumulative cleavage of different CSPG as previously mentioned [38, 95]. Nevertheless, given that aggrecan is one of the main component of PNNs, we can hypothesize that plasmin is more likely to influence PNN remodeling by acting on this particular CSPG. Indeed, aggrecan was shown to play a crucial role in PNN structure since its conditional knockout in the brain results in complete PNN loss in the cortex and the reactivation of juvenile plasticity [72]. Interestingly, both tPA and plasmin are also involved in plasticity-related mechanisms such as ocular dominance shift and dendritic spine remodeling during the critical period [52, 53]. In view of these results, we hypothesize that PNN degradation by the tPA/plasmin system could promote the restoration of juvenile plasticity under pathological conditions.

## Conclusions

In summary, our findings show that in the presence of plasminogen, tPA released from FS-PV cells reduces PNN density through CSPG degradation. This could affect several PNN properties, including balancing GABAergic and glutamatergic neurotransmissions [2, 22, 42, 70, 76, 78], being a physical protective barrier [10, 29, 57], a contributor to pathogenic pathways [40, 49, 62, 97], and a driver of synaptic plasticity and behavioral outcome [11, 29, 70, 76]. Overall, we provide unanticipated mechanistic insights in the regulation of PNNs with relevance to neuronal function, which could translate into new targets to promote plasticity/recovery under pathological conditions.

## Methods

### Animals

All experiments were conducted in accordance with the French ethical law (Decree 2013-118) and the European Communities Council guidelines (2010/63/EU). Protocols were approved by our local ethics committee dependent on the French Ministry of Research and Higher Education (agreement numbers Cenomexa #25267 and Ce5/2012/062). All applicable international, national, and/or institutional guidelines for the care and use of animals were followed. Electrophysiological experiments were performed on G42 transgenic mice (Jackson laboratories #007677, GAD67-GFP, 50). Histological analyses were performed on 8-week-old male Swiss mice, tPAFlox^+/+^ mice, tPAFlox^+/+^; PV-Cre^+/−^ mice and their control (tPAFlox^−/−^; PV-Cre^+/−^) littermates (20-25g). Pregnant tPA Null mice and their WT littermates at gestational day 14 were used for in vitro neuronal cultures.

Animals were housed with a 12-h light/12-h dark cycle with free access to water and food.

tPA Null mice were generated by the Mouse Clinical Institute (ICS, Illkrich, France). Briefly tPAFlox^+/+^ mice (on a C57BL6NTac genetic background) in which exon 3 is flanked by loxP sites (see 3) were crossed with Rosa^26^-Cre mice [7] to induce Cre-mediated excision of the third exon in germline. Mice were genotyped by PCR analysis and southern blots, using tail genomic DNA samples, to detect the presence/absence of loxP sequences. PV-Cre^+/−^ female mice (Jackson laboratory # 008069, Pvalbtm1(cre)Arbr, [33]) were crossed with tPAFlox^+/+^ male mice or tPAFlox^−/−^ male mice to generate tPAFlox^+/+^; PV-Cre^+/−^ (cKO) and their control littermates tPAFlox^−/−^; PV-Cre^+/−^. Mice were genotyped by PCR for the presence of loxP sites and Cre transgene.

### Whole-cell recordings in acute slices

G42 mice were deeply anesthetized with isoflurane. After decapitation, brains were quickly removed and placed into ice-cold oxygenated artificial cerebrospinal fluid (aCSF) containing (in mM): 126 NaCl, 2.5 KCl, 1.25 NaH_2_PO_4_, 2 CaCl_2_, 1 MgCl_2_, 26 NaHCO_3_, 10 glucose, 15 sucrose, and 1 kynurenic acid (Sigma). Coronal slices (300 μm thick) containing the primary visual cortex were cut with a vibratome (VT1000S; Leica) and allowed to recover at room temperature for at least 30 min in aCSF saturated with O_2_/CO_2_ (95 %/5 %). Patch pipettes (4–6 MΩ) pulled from borosilicate glass were filled with 8 μl of RNAse-free internal solution containing in mM: 144 K-gluconate, 3 MgCl_2_, 0.5 EGTA, 10 HEPES, pH 7.2 (285/295 mOsm). Whole-cell recordings were performed at room temperature using a patch-clamp amplifier (MultiClamp *700B*, MDS). Membrane potentials were not corrected for liquid junction potential. Data were filtered at 10 kHz and digitized at 20 kHz using an acquisition board (Digidata 1440, MDS). Cells were set at −60 mV by continuous current injection and submitted to current pulses (800 ms, from –100 to 280 pA with 20 pA increments).

### Cytoplasm harvesting and scRT-PCR

At the end of the whole-cell recording, lasting <15 min, the cytoplasmic content was harvested in the recording pipette. The content of the pipette was expelled into a test tube and RT was performed in a final volume of 10 μL, as described previously [39]. The scRT-PCR protocol was designed to probe simultaneously the expression of neuronal markers and key molecular elements including tissue-type plasminogen activator gene, Plat. Two-step amplification was performed essentially as described [13, 20]. Briefly, cDNAs present in the 10 μL reverse transcription reaction were first amplified simultaneously using all external primer pairs (Additional file 1: Table S1). Taq polymerase (2.5 U; Qiagen) and 20 pmol of each primer were added to the buffer supplied by the manufacturer (final volume, 100 μl), and 20 cycles (94°C, 30 s; 60°C, 30 s; and 72°C, 35 s) of PCR were run. Second rounds of PCR were performed using 1 μl of the first PCR product as a template. In this second round, each amplicon was individually re-amplified using its specific nested primer pair (Additional file 1: Table S1) by performing 35 PCR cycles as described above. Ten microliters of each individual PCR product was run on a 2 % agarose gel stained with ethidium bromide using ΦX174 digested by *Hae*III as a molecular weight marker.

### Unsupervised clustering

To classify cells, unsupervised clustering was performed using 16 molecular parameters (VGlut1, Gad65, Gad67, PV, Akr1c18, Sst, S100β, APC, Mybpc1, Ncan, Plat, Plau, Ptprr, Ptprz1, Sdc4, Sema3a). The presence of a given gene was digitized by 1 and its absence was digitized by 0. Ward-linkage hierarchical clustering [92] was performed and plotted as a dendrogram using “scipy.cluster.hierarchy” functions on Python 3.7. The final number of clusters was suggested by the Thorndike procedure [88] as described previously [14].

### Viral production

Viral particles were provided by Gilles Bonvento and Alexis Bemelmans (INSERM U1169/MIRCen CEA, Fontenay aux Roses 92265, France).

The clone encoding for a GFP under the control of a 1.2-kb sequence of the Human Plat promoter (spanning from −1035 to +207 bp from the transcription start codon) was purchased from GeneCopoeia (pPlat-GFP; Catalog No.: HPRM12655-PF02). The pPlat-GFP was then subcloned in the pDONR221 for AAV production. All the constructs were amplified in *Escherichia coli* JM109 cells and purified by a Nucleobond endotoxin-free plasmid DNA PC 2000 kit (Macherey-Nagel) according to the manufacturer’s instructions. The vector used for this study was an AAV9 serotype. Self-complementary AAV vectors expressing the Plat-GFP construct were produced by transfecting HEK293 cells with the adenovirus helper plasmid (pXX6-80), the AAV packaging plasmid carrying the rep2 and the cap8 genes, and the AAV2 shuttle plasmid containing the Plat-GFP transgene in a sc genome. Recombinant vectors (rAAV) were purified by ultracentrifugation on a discontinuous iodixaniol density gradient followed by dialysis against the formulation buffer of the vector stocks, 0.5 mmol/l MgCl_2_ and 1.25 mmol/l KCl in phosphate-buffered saline (PBSMK; five buffer changes, 3 h per round of dialysis). Physical particles were quantified by real-time PCR. Vector titers are expressed as viral genomes per milliliter (vg/mL).

### Stereotaxic injection of AAV virus

Animals were deeply anesthetized with isoflurane 5 % and maintained with 2 % isoflurane in a 70 %/30 % mixture of NO_2_/O_2_ in a stereotaxic frame (Havard Apparatus). AAV9-Plat-GFP (1.25.10^13^ vp/mL) were injected through a glass micropipette in the right hemisphere in a volume of 0.5 μL at a rate of 0.2 μL/min. Coordinates (relative to bregma) according to the Paxinos Mouse Brain Atlas were as follows: AP: −0.25 mm; ML: −3.4 mm; DV: −0.4 mm and −0.8 mm for the somatosensory cortex. The needle was left in position for a further 5 min and then removed slowly from the brain. After recovery from surgery, mice were left undisturbed for 3 weeks for effective and stable transgene expression.

### Antibodies

The following antibodies, lectins, and biotinylated protein were used: mouse anti-NeuN (1:800, MAB377, Merck); anti-GFP (rabbit, 1:1000, ab6556, abcam or chicken, 1:1000, ab13970, abcam); chicken anti-Glial Fibrillary Acidic Protein (GFAP) (1:2000, ab4674, abcam); rabbit anti-ionized calcium-binding adapter molecule-1 (Iba-1) (1:1000, 019-19741, Fujifilm); rabbit anti-GABA (1:500, A2052, Sigma Aldrich); anti-parvalbumin (rabbit, 1:3000, ab 11427, abcam or guinea pig, 1:500, GP72, Swant); rabbit anti-aggrecan (1:1000, AB1031, Merck); or Biotinylated Wisteria floribunda Agglutinin (WFA) (1:1000, L1516, Sigma Aldrich).

### Immunohistochemistry

Mice were deeply anesthetized with isoflurane 5 % in 70 %/30 % mixture of NO_2_/O_2_. A transcardial perfusion was performed with ice-cold 0.9 % NaCl with 3 % heparin followed by 150 ml of fixative solution containing 4 % paraformaldehyde (in PBS 0.1M, pH 7.4). Brains were removed and cryoprotected in 20 % sucrose solution (in PBS 0.1M, pH 7.4) for 24h and frozen in Tissue-Tek (Miles Scientific). Cryostat sections of 10 μm were collected on Poly-Lysine slides and stored at −80°C. Sections were incubated overnight at room temperature with primary antibodies. Corresponding Fab’2-conjugated secondary antibodies were diluted at 1:800 (Jackson Immunoresearch). Images were acquired using Leica DM6000 microscope-coupled CoolSnap camera, visualized with Metamorph 7.0 software (Molecular Devices), and further processed were realized using ImageJ software.

For 3D reconstruction, images were acquired using Leica TCS SP8 Confocal/STED 3 × microscope with an oil-immersion ×40, 1.44-N.A. objective at a resolution of 512×512 pixels with 572 Hz speed and a step size of 0.22μm.

### Histological analysis

To estimate the percentage of each Plat-GFP cell, all transfected cells were quantified (1090.3 ± 58.9 Plat-GFP cells/brain, from four sections per brain, 3 WT mice). For PNN/PV cells quantification, 2 images per section were acquired to cover all layers of the somatosensory cortex with a good resolution (four sections per brain, five mice per condition). PNN intensity was calculated as the intensity of WFA staining through the entire section of the somatosensory cortex. The overall intensity of WFA staining considers the high WFA staining around the soma and proximal dendrites and low WFA staining found around dendrites. PNN morphology was evaluated using a qualitative classification, ranging from High+ (strong WFA staining) to Low− (faint WFA staining). Only cells of which the soma was in the focal plane were classified. Analyses were performed blind to genotype.

### Primary cultures of interneurons

GABAergic interneurons derived from the medial ganglionic eminence (MGE) were prepared from fetal mice (embryonic day 14) as previously described [27]. Medial ganglionic eminences were dissected in HBSS (Hanks’ Balanced Salt Solution, Gibco) /HEPES 10mM, incubated 15 min at 37°C in HBSS/HEPES with trypsin (0.05 %, Gibco) and DNAse I (100 μg/mL, Worthington) and mechanically dissociated through a glass pipette. Then, cells were plated (425,000 cells/mL) on glass bottom microwell dishes (MatTek Corporation) or on 24-well plates (500,000 cells/mL) previously coated with poly-d-lysine (0.1 mg/mL, Sigma Aldrich, P6407-5MG) and laminin (0.02 mg/mL, Gibco) in Neurobasal medium (Life Technologies) containing 2 % B27 supplement (Life Technologies) and 1 % Glutamax (Life Technology). Cytosine β-D-arabinofuranoside hydrochloride (5 μM, Sigma Aldrich) was added at DIV3, and fresh medium was added at DIV7.

### In vitro treatments

Cells were treated at DIV14 with either 100 nM Human Glu-plasminogen (Enzyme Research Laboratories, HPg 2001), 200 nM Human Plasmin (Enzyme Research Laboratories, HPlasmin), or 10 nM or 200 nM Human tPA (Actilyse®, Boehringer Ingelheim) for 24 h.

### Immunocytochemistry

Cells were fixed during 10 min in 0.1 M PBS containing 4 % paraformaldehyde. After PBS washes, cells incubated for 1 h in PBS-Triton 0.25 % containing 1 % BSA and then incubated overnight at 4 °C in blocking buffer containing primary antibodies. Confocal images were acquired with a Leica TCS SP8 Confocal/STED 3 × microscope with an oil-immersion ×40, 1.44-N.A. objective. Confocal images were taken at a resolution of 1024×1024 pixels with 400 Hz speed and a step size of 0.45μm. Laser intensity, gain, and offset were maintained constant in each analysis.

### PNN quantification in vitro

Confocal images were analyzed with the ImageJ software. Random PV-positive cells were imaged with 160 × 160 × 15 μm z-stack with 0.45 μm step size. A threshold was applied to the WFA channel or aggrecan channel (range 0–255), and the area was quantified in 40 neurons from 4 independent experiments.

### Enzymatic degradation assays

Recombinant human aggrecan core protein G1-IGD-G2 (156 nM) (Biotechne) was incubated at 37°C overnight with either 7.8 nM recombinant human ADAMTS4 protein (Biotechne), purified human plasmin (Enzyme Research Laboratories), Human Glu-plasminogen (Enzyme Research Laboratories), and/or tPA (Actilyse) in a buffer containing 50 mM Tris, 10mM CaCl_2_, 150 mM NaCl, and 0.05 % Brij35. Then, 78 nM Aprotinin treatment was applied with either Glu-plasminogen + tPA treatment or plasmin treatment. Digestion products were analyzed by SDS-PAGE on 10 % Tris-Glycine gel and stained with GelCode Imperial Blue Stain Reagent (Pierce).

### Statistics

For sc-PCR analyses, between-group comparisons were performed using Mann–Whitney nonparametric test. Comparison of the occurrence of expressed genes between cell types was determined using Fisher’s exact test. A *p*-value below 0.05 was considered statistically significant.

Otherwise, experiments were analyzed by investigators blinded to group allocation. Unblinding was made after completion of statistical analysis. All statistical analyses were performed using the GraphPad Prism 8 software. Comparisons between more than two independents samples were performed using two-tailed Kruskal-Wallis test and appropriate post hoc test (Dunn’s test). Mann–Whitney *U* test was used for the comparisons of two independent samples. Plasmin activity was analyzed using 2-way Anova (two-stage linear step-up procedure of Benjamini, Krieger and Yekutieli). Recombinant aggrecan degradation was analyzed using one-way ANOVA followed by Fisher’s LSD test. The intensity of the 120-kDa band was compared between Agg G1-G2 and protease-treated conditions. A threshold of *P* < 0.05 was defined as statistically significant. All graph data are presented as the mean standard error mean (SEM). Sample sizes are indicated in each corresponding legend.

## Supplementary Information

**Additional file 1: Figure S1.** Characterization of a tPA-reporter viral construct in the dentate gyrus. **Figure S2.** Plat-GFP reporter expression in the cortex after LPS treatment. **Figure S3.** Plat-GFP reporter expression in excitatory neurons in the cortex. **Figure S4.** tPA mRNA is expressed in PV interneurons enwrapped with PNNs. **Figure S5.** Conditional depletion of tPA in PV interneurons. **Figure S6.** PNN-like structures *in vitro*. **Figure S7.** Interneuron-derived tPA promotes PNN degradation through plasmin. **Figure S8.** tPA/plasmin mediated PNNs degradation is MMP-independent. **Table S1.** Sequences of PCR primers. **Supplementary materials and methods**.

## Data Availability

All data generated or analyzed during this study are included in this published article and its supplementary information files. All data supporting our results are available from the corresponding author upon reasonable request.

## Abbreviations

AAV: Adeno-associated viruses
ADAMTS: A Disintegrin And Metalloproteinase with Thrombospondin Motifs
CSPGs: Chondroitin sulfate proteoglycans
ECM: Extracellular matrix
FS-PV: Fast-spiking parvalbumin
GABA: Gamma-aminobutyric acid
LRPs: Low-density lipoprotein receptor–related proteins
MMPs: Matrix metalloproteinases
NMDAR: N-methyl-D-aspartate receptor
PNN: Perineuronal nets
PV: Parvalbumin
TIMP3: Tissue inhibitor of metalloproteinases-3
tPA: Tissue-type plasminogen activator
VIP: Vasointestinal peptide
WFA: Wisteria floribunda agglutinin
