# Supplementary material for: Parvalbumin interneuron-derived tissue-type plasminogen activator shapes perineuronal net structure
Source: BMC Biol. 2022 Oct 5;20:218. doi: 10.1186/s12915-022-01419-8 (PMC9535866; doi:10.1186/s12915-022-01419-8)
Supplement: Supplementary file 1 — Additional file 1: Figure S1. Characterization of a tPA-reporter viral construct in the dentate gyrus. Figure S2. Plat-GFP reporter expression in the cortex after LPS treatment. Figure S3. Plat-GFP reporter expression in excitatory neurons in the cortex. Figure S4. tPA mRNA is expressed in PV interneurons enwrapped with PNNs. Figure S5. Conditional depletion of tPA in PV interneurons. Figure S6. PNN-like structures in vitro. Figure S7. Interneuron-derived tPA promotes PNN degradation through plasmin. Figure S8. tPA/plasmin mediated PNNs degradation is MMP-independent. Table S1. Sequences of PCR primers. Supplementary materials and methods. [file 12915_2022_1419_MOESM1_ESM.docx]

Additional File 1

**Parvalbumin interneuron-derived Tissue-type plasminogen activator shapes perineuronal net structure**

Matthieu Lépine^a1^, Sara Douceau^a1^, Gabrielle Devienne^b^, Paul Prunotto^a^, Sophie Lenoir^a^ Caroline Regnauld^a^, Elsa Pouettre^a^, Juliette Piquet^b^, Laurent Lebouvier^a^, Yannick Hommet^a^, Eric Maubert^a^, Véronique Agin^a^, Bertrand Lambolez^b^, Bruno Cauli^b^, Carine Ali*****^a1^, Denis Vivien^a,c1^

^a^ Normandie Univ, UNICAEN, INSERM, INSERM UMR-S U1237, Physiopathology and Imaging of Neurological Disorders, Institut Blood and Brain @ Caen-Normandie, Cyceron, Caen, 14000, France.

^b^ Neuroscience Paris Seine - Institut de Biologie Paris Seine (NPS - IBPS), Sorbonne Université UM119, CNRS UMR8246, INSERM U1130, 75005 Paris, France

^c^ Department of clinical research, CHU de Caen Normandie, Caen, France

^1^ Authors with equal contribution

***Corresponding author. Email: ali@cyceron.fr**

**This file include:**

Figure S1 to S8

Table S1

Supplementary materials and methods

**
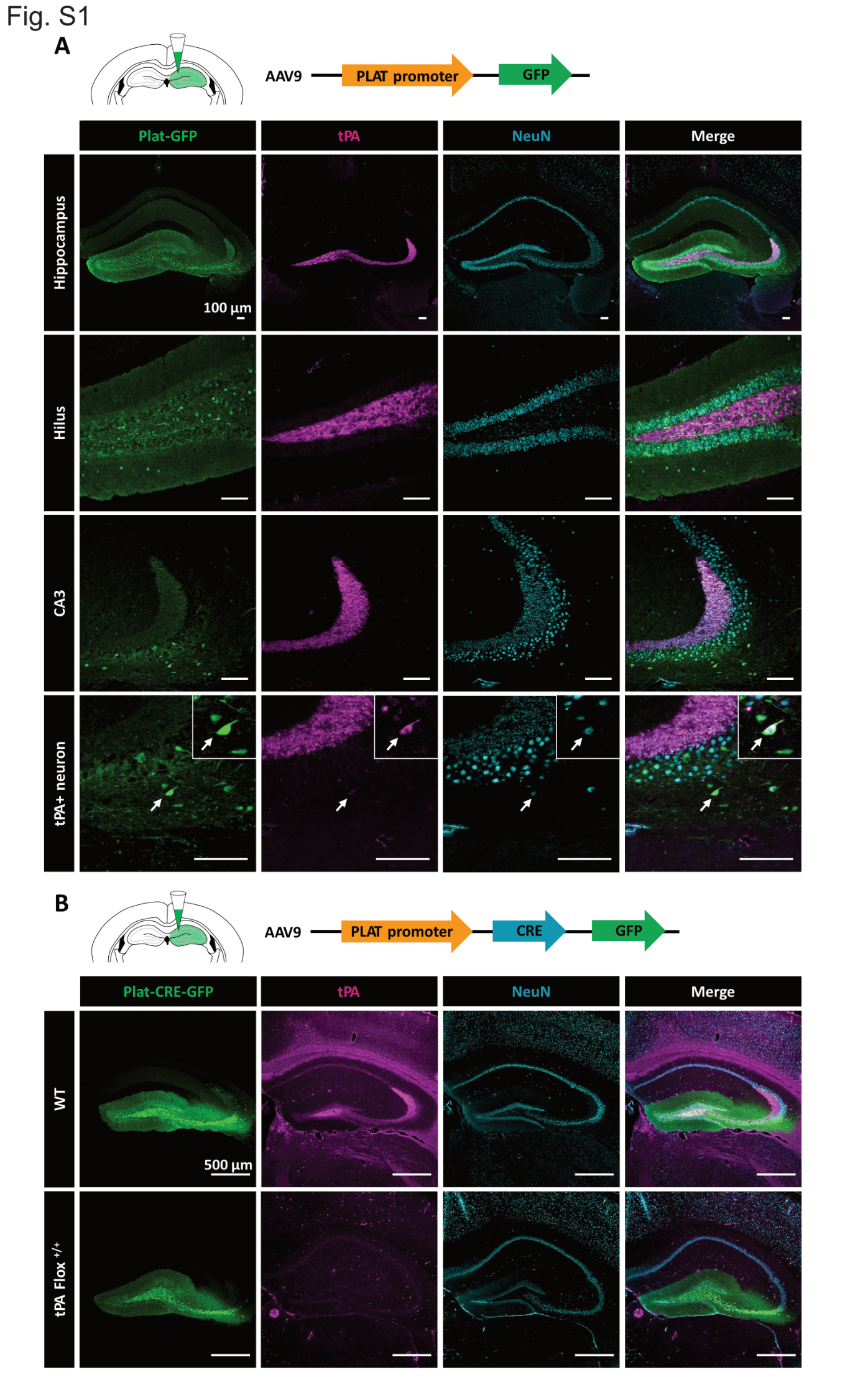
**

**Figure S1: Characterization of a tPA-reporter construct, for *in vivo* investigations – AAV9-Plat-GFP**

(A) We engineered a viral reporter construct encoding for green fluorescent protein (GFP) under the control of the human tPA (Plat) promoter (-1035 to +207 from the transcription start site; AAV-Plat-GFP; see Methods section). The construct was first injected in the dentate gyrus (DG), a brain structure in which tPA immunostaining is well described [42,44]. Co-Immunohistochemistry of the GFP reporter (green) with the neuronal marker NeuN (cyan) and tPA (magenta) were performed in the hippocampus of adult wild type mice, 3 weeks after injection. The GFP staining was observed in the granular cells of the DG, in mossy cells of the hilus and in mossy fibers in the CA3 sub-region of the hippocampus. Hippocampal GFP signals in mossy fibers co-localized with signals from immunostaining for a tPA-specific antibody [42].

(B) To validate the specificity of the Plat-GFP construct, tPAFlox^+/+^ mice [24] were injected in the DG with an AAV-Plat-Cre-GFP construct (See Methods section). Co-immunohistochemistry of the GFP reporter (green) with the neuronal marker NeuN (cyan) and tPA (magenta) in the hippocampus 3 weeks after injection. As expected, Cre-GFP was detected in both granular cells of the DG and in the corresponding mossy fibers , while co-immunostaining for tPA was negative, confirming the efficient and specific conditional knock-out in tPAFlox^+/+^ mice but not in their wild type littermates. Thus, the Plat-GFP construct is a valid reporter of tPA expression *in vivo*. Scale bar: 100 µm.

**
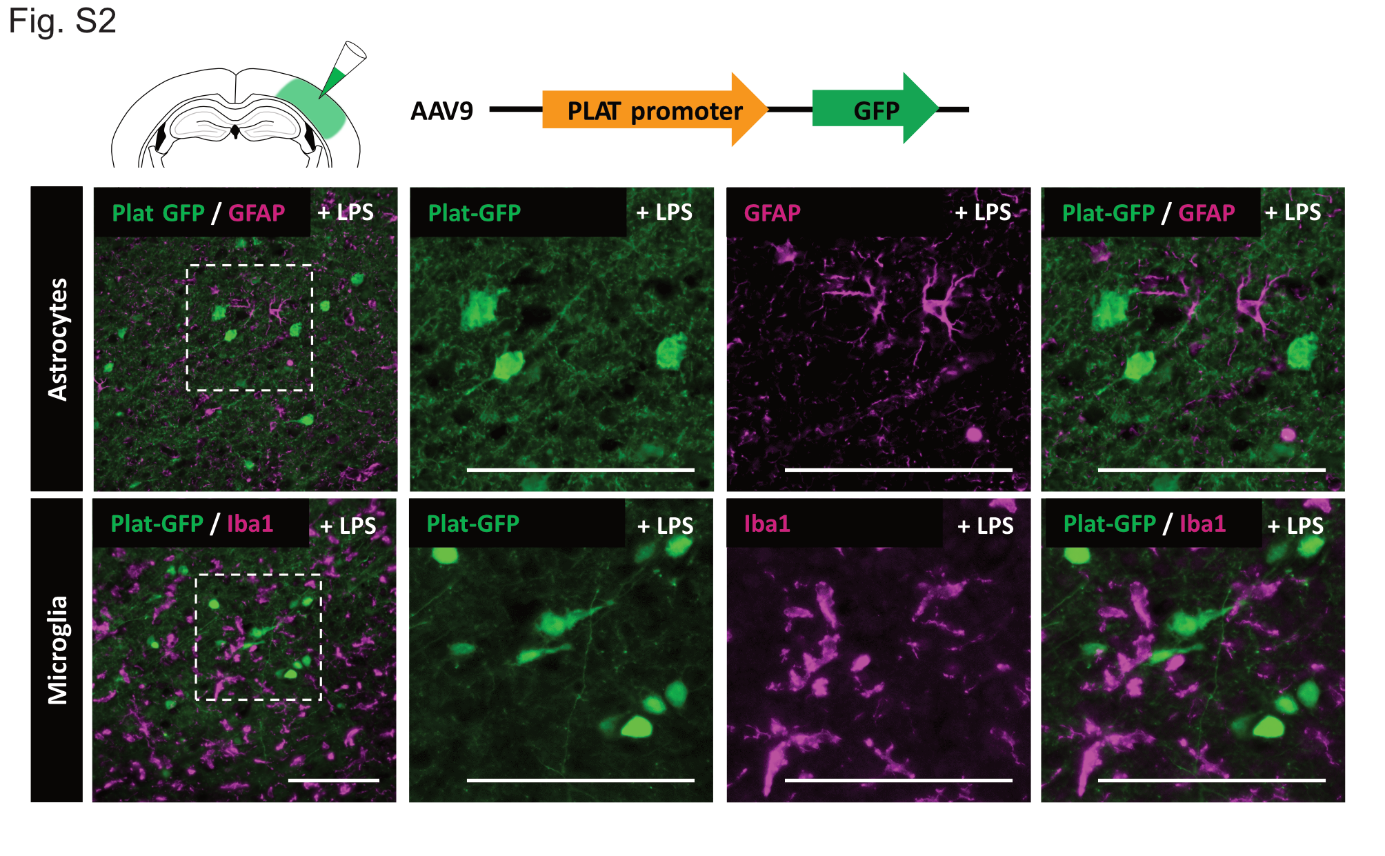
**

**Figure S2: Plat-GFP expression in the somatosensory cortex of LPS-treated mice**

AAV-Plat-GFP was injected in the somatosensory cortex of adult wild-type mice. Co-immunohistochemistry of the GFP reporter (green) with the astrocyte marker GFAP (magenta) and the microglial marker Iba-1 (magenta) in the somatosensory cortex of adult WT mice under inflammatory conditions (LPS injection in the somatosensory cortex). Scale bar: 100 µm.

**
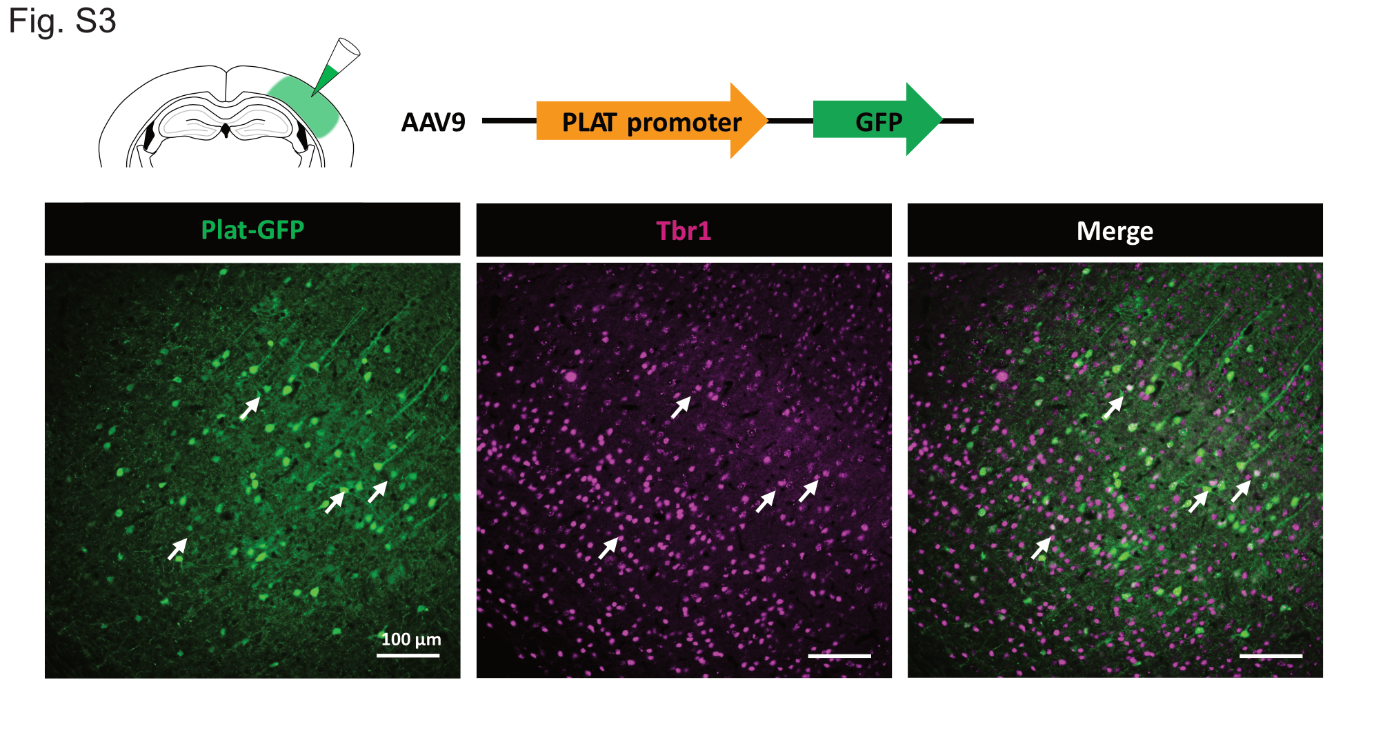
**

**Figure S3: Plat-GFP expression in excitatory neurons in the somatosensory cortex**

AAV-Plat-GFP was injected in the somatosensory cortex of adult WT mice. Immunohistochemistry of the GFP reporter (green) with the excitatory neuronal marker Tbr1 (magenta) shows a localization of Plat-GFP in excitatory neurons. Scale bar: 100 µm.

**
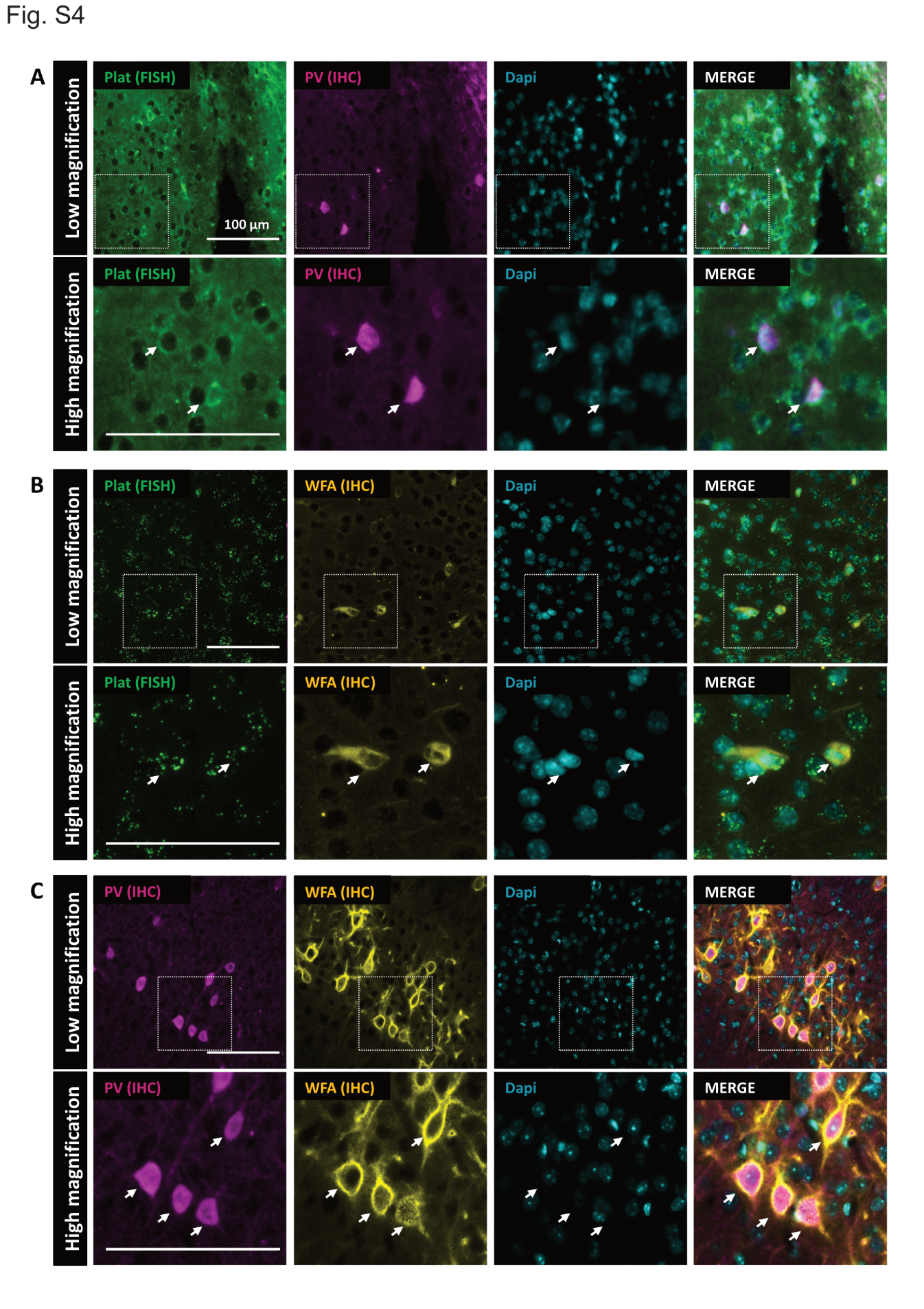
**

**Figure S4: tPA mRNA is expressed in parvalbumin interneurons enwrapped with PNNs**

(A-B) *In situ* hybridization against tPA mRNA (Plat; green dots) in the somatosensory cortex of WT mice, revealing tPA mRNA detection in parvalbumin interneurons (magenta) and in neurons enwrapped by PNNs (WFA, yellow). (C) Immunohistochemistry in the somatosensory cortex, revealing PNN marker (WFA) mostly around parvalbumin positive cells. Scale bar: 100 µm.

**
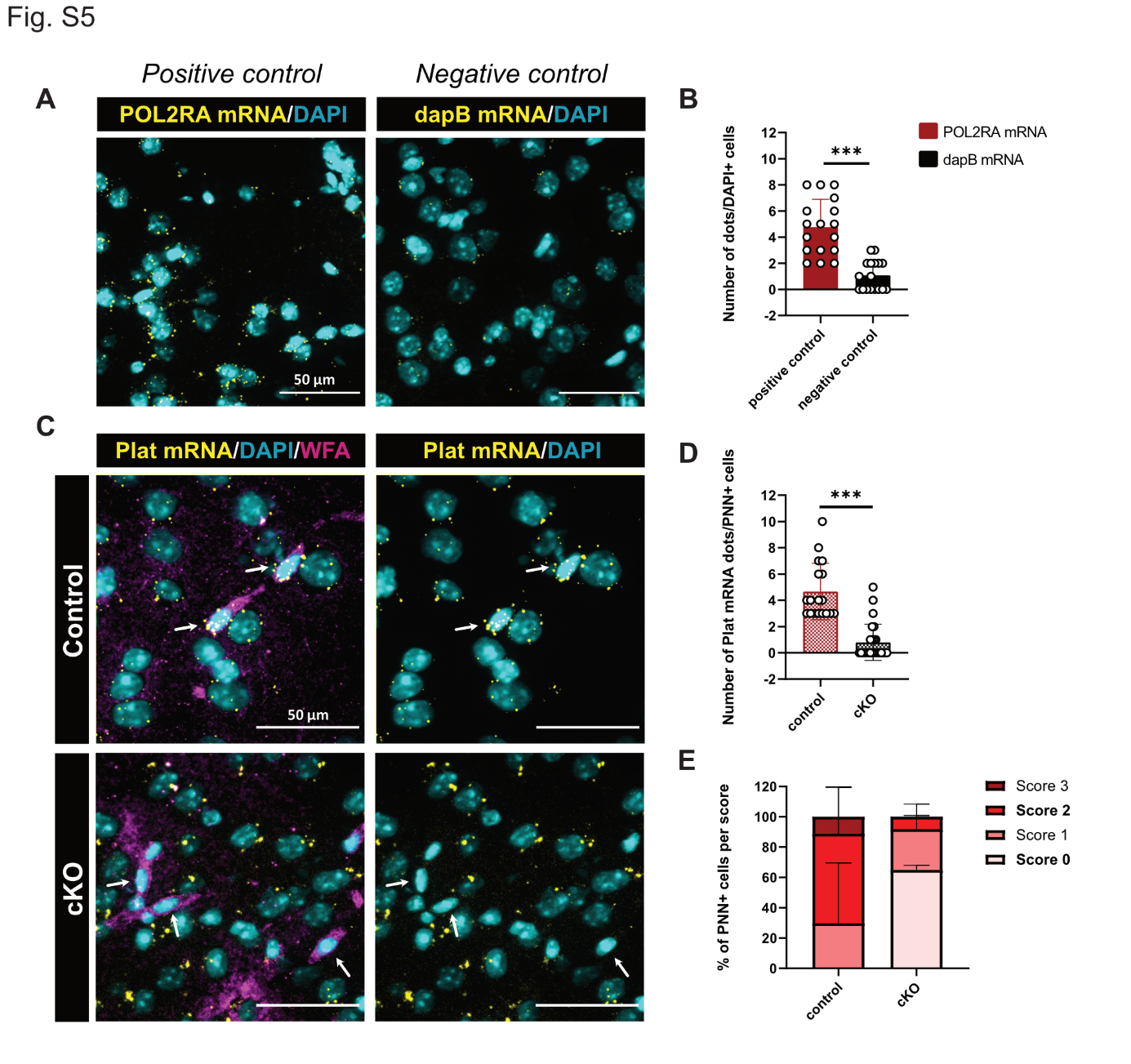
**

**Figure S5: Endogenous tPA mRNA is expressed by PNN positive cells in WT mice but not in mice with a selective deletion of tPA in PV.**

(A) Representative images of RNAscope positive control POL2RA mRNA (yellow) and negative control dapB mRNA (yellow). (B) Quantification of the number of mRNA dots found in each condition. N=17 DAPI+ cells per condition. Mann Whitney test; p<0.001. (C) Representative images showing Plat mRNA dots (yellow) in PNN+ cells (magenta) in control mice and cKO mice. Note that Plat mRNA dots are still present in PNN- cells in cKO condition. (D) Quantification of the number of Plat mRNA dots found in Control and cKO mice. Control: N=18 PNN+ cells from 3 control mice; cKO: N= 25 PNN+ cells from 3 cKO mice. Mann Whitney test; p<0.001. (E) Quantification of the percentage of cells per score in control mice and cKO mice. N=3 mice per genotype. Two-way ANOVA (score*genotype: p<0.01); Sidak post-hoc test: score 2: p<0.05; score 0: p<0.01. Scale bar = 50 µm. Graph show mean ± sem.

**
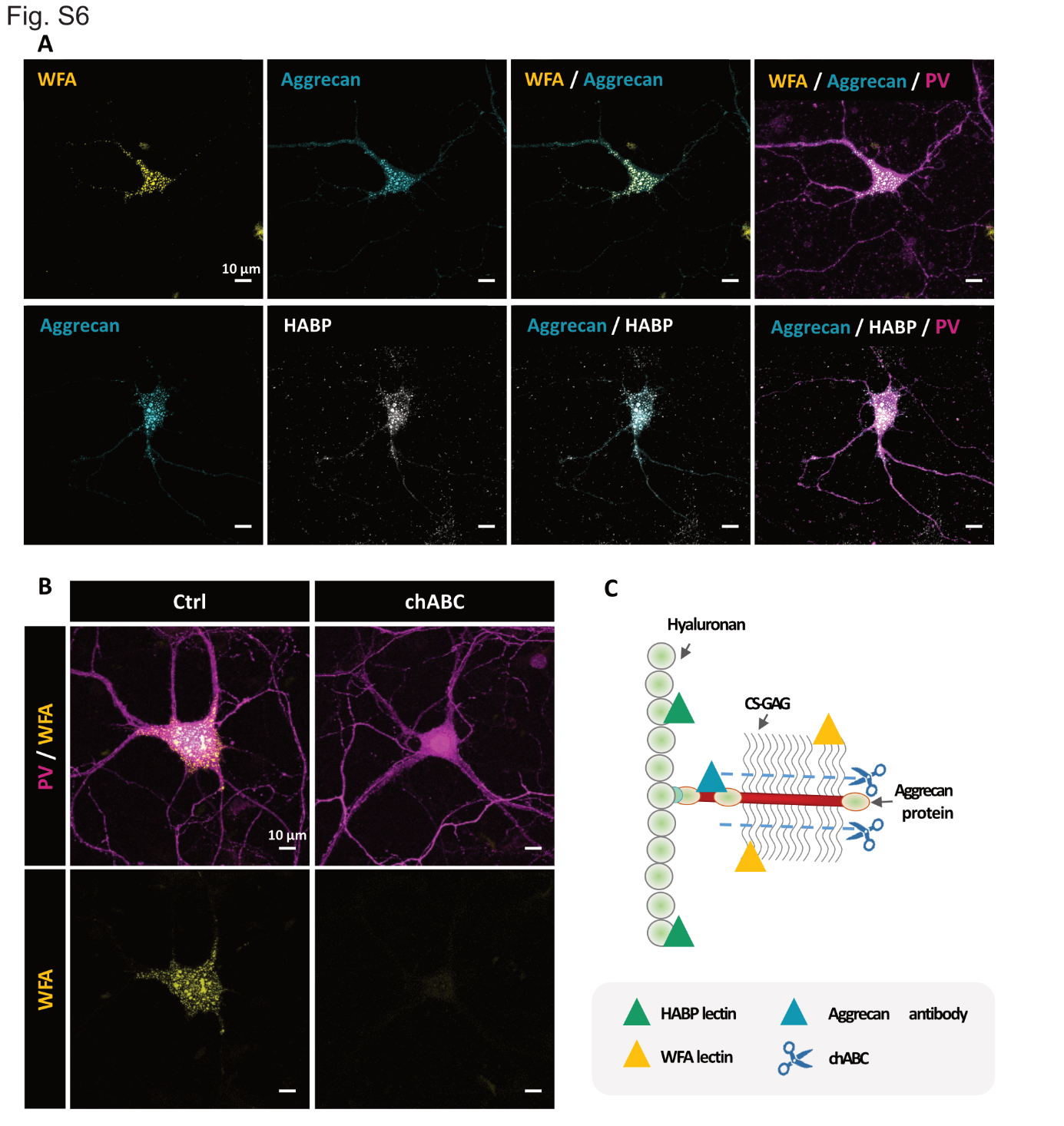
**

**Figure S6: PNN-like structures express aggrecan and hyaluronic acid and are sensitive to ChABC treatment**

(A) Neurons at DIV14 stained for WFA (yellow) aggrecan (cyan), HABP (Hyaluronic acid binding protein; white) and PV (magenta). (B) Chondroitinase ABC (ChABC) treatment during 24h enables a complete removal of PNN-like revealed by WFA staining.(C) Schematic representation showing WFA binding to chondroitin-sulfate glycosaminoglycans chains (CS-GAG) of CSPG, HABP binding to hyaluronan and ChABC degradation of CS-GAG. Scale bar: 10 µm.

**
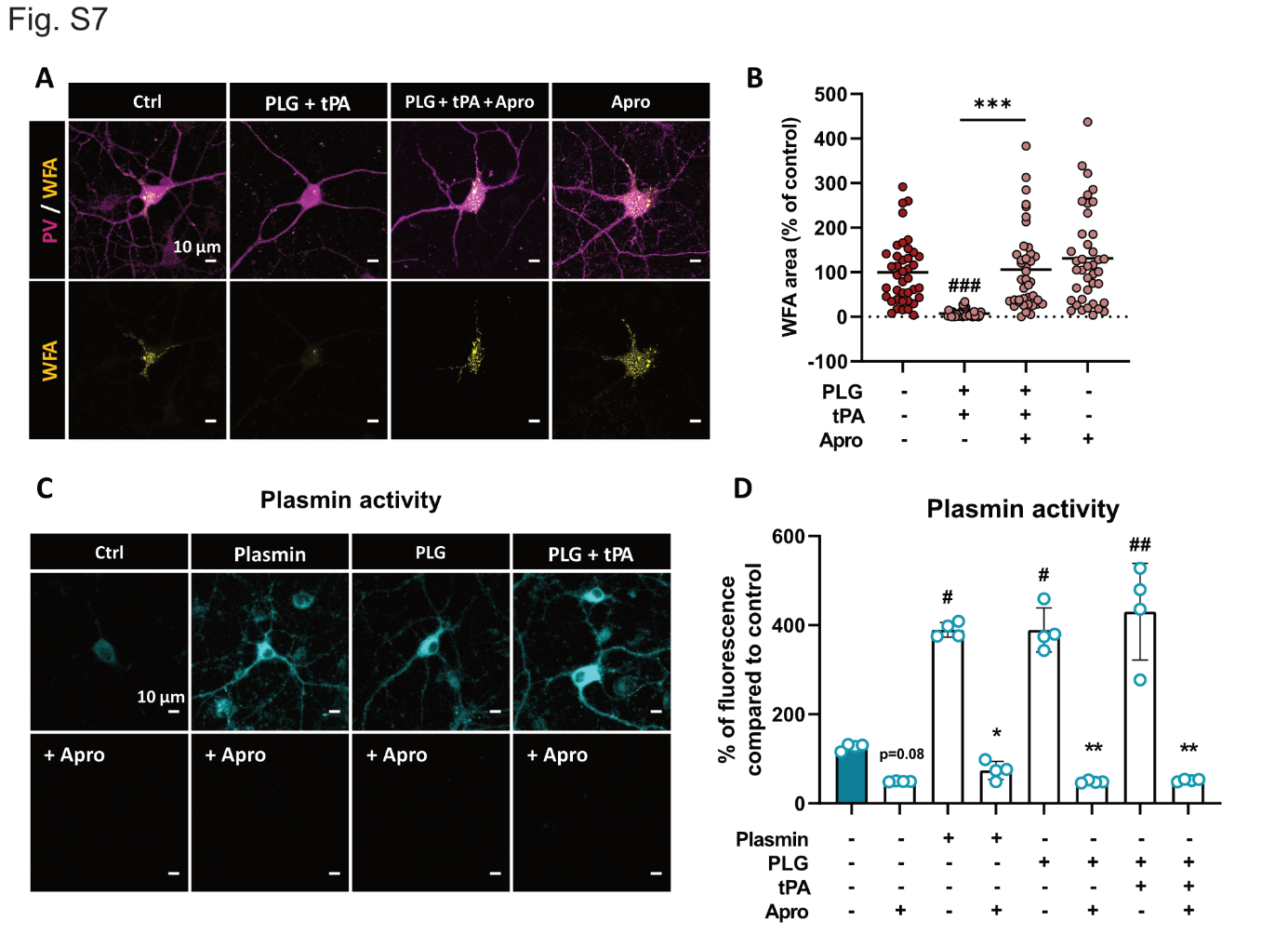
Figure S7: Endogenous tPA controls the remodeling of PNNs in a plasmin- dependent manner**

(A-B) MGE-derived WT interneuron cultures were treated at DIV14 with plasminogen (100 nM), tPA (10 nM) and aprotinin (1.5 µM) and processed 24h later for immunocytochemistry. (A) Representative images of immunocytochemical stainings for PV (magenta) and WFA (yellow) 24h after treatment. (B) Aprotinin reverses plasminogen + tPA-induced PNN-like degradation. Graph show mean ± sem (n=40 cells from 4 independent experiments). Kruskall-Wallis test followed by Dunn’s post-hoc test for multiple comparisons; ###: p<0.001 (compared to Control), ***: p<0.001 (compared to PLG + tPA).(C-D) Plasmin activity was assessed by measuring the fluorescence emission of a plasmin substrate at 500 nm following treatments of neuronal cultures with plasmin (positive control), plasminogen (PLG), tPA and aprotinin (Apro). Graph show means ± sem (n=4 independent experiments; Kruskall Wallis followed by two-stages linear step-up procedure of Benjamini, Krieger and Yekutieli; #: p<0.05; ##: p<0.01; ###: p<0.001: compared to Control; *: p<0.05; **: p<0.01; ***: p<0.001: compared to respective aprotinin treatment.

**
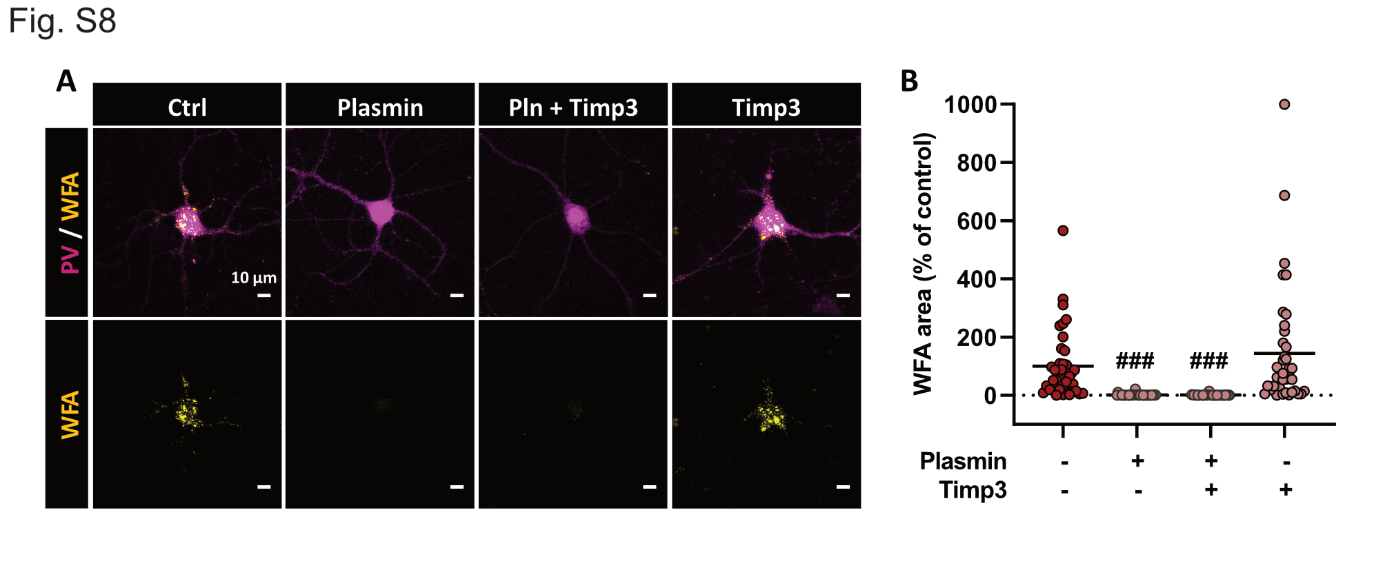
**

**Figure S8: tPA/plasmin mediated PNNs degradation is MMP-independent**

MGE-derived WT interneuron cultures were treated at DIV14 with plasmin (200 nM) and TIMP3 (60 nM) and processed 24h later for immunocytochemistry. (A) Representative images of immunocytochemical stainings for PV (magenta) and WFA (yellow) 24h after treatment. (B) Quantitative analysis of WFA positive area show that plasmin reduces WFA staining whereas TIMP3 treatment has no effect on plasmin-dependent PNNs degradation. Graph show mean ± sem (n=30-40 cells from 4 independent experiments). Kruskall-Wallis test followed by Dunn’s post-hoc test for multiple comparisons; ###: p<0,001 (compared to Control). Scale bar: 10 µm.


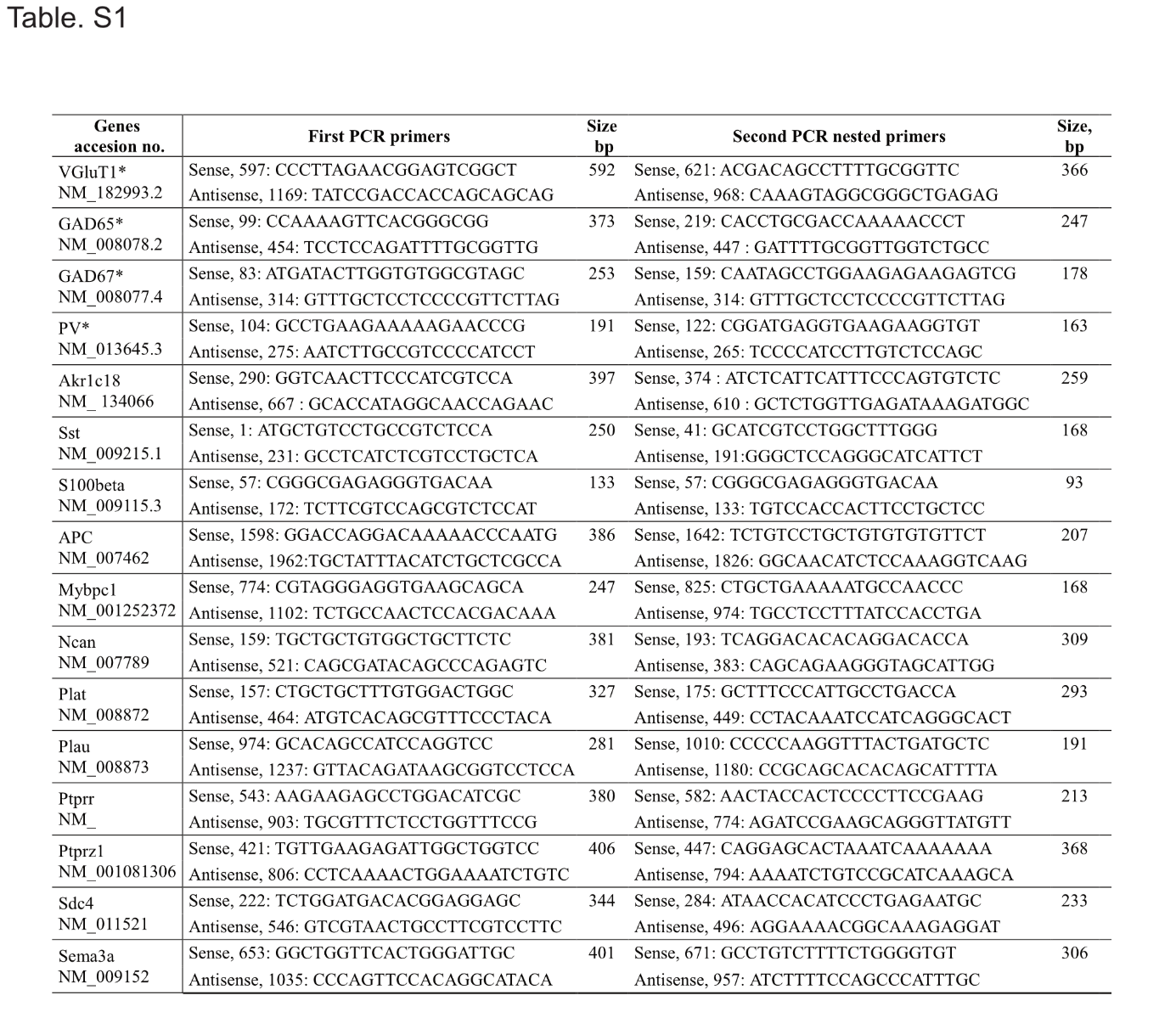


**Table S1: Sequences of first and second PCR primers**

**Supplementary materials and methods**

Viral production:

For the pPlat-Cre-GFP, the cDNA encoding for the Cre recombinase was subcloned by fusion into the pPlat-GFP (In-fusion HD cloning Kit Clontech-Takarabio). The pPlat-Cre-GFP was then subcloned in the pDONR221 for AAV production. All constructs were amplified in Escherichia coli JM109 cells and purified by a Nucleobond endotoxin-free plasmid DNA PC 2000 kit (Macherey-Nagel) according to the manufacturer's instructions.

Stereotaxic injection of AAV virus:

Animals were deeply anesthetized with isoflurane 5 %, and maintained with 2 % isoflurane in a 70 %/30 % mixture of NO_2_/O_2_ in a stereotaxic frame (Harvard Apparatus). AAV9-Plat-GFP or AAV9-Plat-Cre-GFP (1.25.10^13^ vp/mL) were injected through a glass micropipette in the right hemisphere in a volume of 0.5 µL at a rate of 0.2 µL/min. Coordinates (relative to bregma) according to the Paxinos Mouse Brain Atlas were as follows: AP: -2mm; ML: ±1mm; DV: -1.8mm for the dentate gyrus (0.5 µL/site) and AP: -0.25 mm; ML: -3.4 mm; DV: -0.4 mm and -0.8 mm for the somatosensory cortex. The needle was left in position for 5 minutes and then removed slowly. After recovery from surgery, mice were left undisturbed for 3 weeks for effective and stable transgene expression.

Antibodies:

The following antibodies were used: rabbit anti-tPA (1:3000; generous gift from R. Lijnen, Leuven); chicken anti- T-box brain 1 (Tbr1) (1:250, AB2261, Merck). Lectin WFA (1:1000, L1516, Sigma Aldrich). and Biotinylated Hyaluronic Acid Binding Protein (HABP) (1:200, 385911, Merck) were used to stain PNN.

*In vitro* treatments:

Cells were treated at DIV14 with either 0.1 U/mL Chondroitinase ABC (Merck, C2905), 1.5 µM Aprotinin (Sigma Aldrich) or 60 nM recombinant Human TIMP3 (Origene, TP762069) for 24 hours.

Plasmin activity assay:

Plasmin activity was assessed with a specific plasmin fluorescent substrate (Sensolyte AFC Plasmin Activity Assay Kit; Ex/Em=380 nm/500 nm; Anaspec, Fremont, CA, USA). Live imaging was performed using a Leica TCS SP8 Confocal/STED microscope.

*In situ* hybridization:

For fresh tissues: mice were deeply anesthetized with isoflurane 5 % in 70 %/30 % mixture of NO_2_/O_2_. A transcardial perfusion was performed with ice cold 0.9 % NaCl with 3 % heparin and the brain was frozen in isopentane. Cryostat coronal sections of 10 µm were collected on Poly-Lysine slides and dried at least 1 hour at – 20°C. Slides were fixed 30 min with a solution containing 4 % paraformaldehyde (PBS 0.1M, in DEPC water, pH 7.4), then digested 15 min by protease-3. Plat probes were hybridized and amplified (RNAscope®). Following the in situ hybridization steps, PV immunostaining was performed.

For fixed tissues: mice were deeply anesthetized with isoflurane 5 % in 70 %/30 % mixture of NO_2_/O_2_. A transcardial perfusion was performed with ice cold 0.9 % NaCl with 3 % heparin followed by 150 ml of fixative solution containing 4 % paraformaldehyde (in DEPC treated with PBS 0.1M, pH 7.4). Brains were post-fixed for 4h then cryoprotected in 20 % sucrose solution (in PBS 0.1M, in DEPC water, pH 7.4) for 24h and frozen in Tissue-Tek (Miles Scientific). Cryostat coronal sections of 10 µm were collected on Poly-Lysine slides and dried at least 1 hour at – 20°C. Slides were treated by target retrieval reagents for 5min at 100°C and protease-plus digestion for 30min. At this step, Plat probes were hybridized and amplified (RNAscope®). Following the in situ hybridization steps, WFA staining was performed. Image analysis and expression assessment was performed by manual counting using ImageJ software. Only dots up to 1.5 microns were considered as positive. Quantifications were performed by counting the number of Plat+ mRNA dots per PNN+ cells. The semi-quantitative ACD 4-scale score was performed as follows: 0 = No staining or <1 dot/cells; 1 = 1–3 dots/cell; 2 = 4–9 dots/cell; 3 = 10–15 dots/cell. Control probes for the bacterial gene DapB (bacterial dihydrodipicolinate reductase; negative control) and the housekeeping gene PolR2a (RNA polymerase II; positive control) were also performed in WT brains.
